# Genomic and demographic processes differentially influence genetic variation across the human X chromosome

**DOI:** 10.1371/journal.pone.0287609

**Published:** 2023-11-01

**Authors:** Daniel J. Cotter, Timothy H. Webster, Melissa A. Wilson

**Affiliations:** 1 Department of Genetics, Stanford University, Stanford, CA, United States of America; 2 Department of Anthropology, University of Utah, Salt Lake City, UT, United States of America; 3 School of Life Sciences, Arizona State University, Tempe, AZ, United States of America; 4 Center for Evolution and Medicine, Biodesign Institute, Arizona State University, Tempe, AZ, United States of America; University of Iceland, ICELAND

## Abstract

Many forces influence genetic variation across the genome including mutation, recombination, selection, and demography. Increased mutation and recombination both lead to increases in genetic diversity in a region-specific manner, while complex demographic patterns shape patterns of diversity on a more global scale. While these processes act across the entire genome, the X chromosome is particularly interesting because it contains several distinct regions that are subject to different combinations and strengths of these forces: the pseudoautosomal regions (PARs) and the X-transposed region (XTR). The X chromosome thus can serve as a unique model for studying how genetic and demographic forces act in different contexts to shape patterns of observed variation. We therefore sought to explore diversity, divergence, and linkage disequilibrium in each region of the X chromosome using genomic data from 26 human populations. Across populations, we find that both diversity and substitution rate are consistently elevated in PAR1 and the XTR compared to the rest of the X chromosome. In contrast, linkage disequilibrium is lowest in PAR1, consistent with the high recombination rate in this region, and highest in the region of the X chromosome that does not recombine in males. However, linkage disequilibrium in the XTR is intermediate between PAR1 and the autosomes, and much lower than the non-recombining X. Finally, in addition to these global patterns, we also observed variation in ratios of X versus autosomal diversity consistent with population-specific evolutionary history as well. While our results were generally consistent with previous work, two unexpected observations emerged. First, our results suggest that the XTR does not behave like the rest of the recombining X and may need to be evaluated separately in future studies. Second, the different regions of the X chromosome appear to exhibit unique patterns of linked selection across different human populations. Together, our results highlight profound regional differences across the X chromosome, simultaneously making it an ideal system for exploring the action of evolutionary forces as well as necessitating its careful consideration and treatment in genomic analyses.

## Introduction

Genetic variation is influenced by factors that vary across genomic regions. Mutation rate [1–5] and recombination rate [6–11] both fluctuate across the genome and differ between sexes. New mutations increase diversity by introducing novel variation, and the frequency at which these mutations occur, or are removed, contributes to observed patterns of variation. Elevated recombination can increase genetic variation by reducing linkage disequilibrium (LD) and thereby reducing rates of background selection and genetic hitchhiking [12, 13]. If recombination affects the local mutation rate via double strand breaks, genetic variation will also be increased [14].

Some regions of the genome (e.g., the X chromosome, the Y chromosome, and the mitochondria) differ in genetic diversity due to differences in effective population size [15]. Under the infinite sites model, expected nucleotide diversity for diploid organisms is 4N_e_μ [16], where N_e_ is the effective population size and μ is the mutation rate. Because diversity is a function of population size, regions of the genome that have a lower N_e_ are expected to have proportionally lower genetic diversity. The X chromosome, in particular, is composed of multiple regions that differ in N_e_. The pseudoautosomal regions exist on both the X and the Y and therefore have a similar N_e_ to that of the autosomes, while the non-pseudoautosomal regions of the X chromosome exist in two copies in females and one copy in males, effectively resulting in ¾ the effective size of the autosomes, assuming random mating. Thus, one would expect differences in genetic diversity across the X chromosome simply due to differences in N_e_.

Selection can also shape genetic diversity across the genome. Linked selection reduces diversity in neutral regions that are closely linked to genes [17, 18] and this effect can be more or less pronounced under differing strengths of linked selection. The effects of background selection and genetic hitchhiking are reduced moving away from selected regions, which leads to an expected increase in diversity with increasing distance from these regions. Consistent with this, diversity increases with distance from genes on both the autosomes and X chromosome [19–21]. Further, the ratio of X to autosome diversity increases with increasing distance from genes [19–21], suggesting that linked selection is stronger on the X chromosome than the autosomes. This could be due to reduced recombination on the X chromosome in genetic males, where X-linked recombination is limited to the pseudoautosomal regions (PARs) or because the X chromosome is hemizygous in males and recessive alleles are thus directly exposed to selection, leading to a disproportionate reduction in diversity in and around genes [13, 22–24].

In addition to the processes described above, patterns of human demography strongly affect patterns of genetic variation [25–27]. For example, African populations generally have higher genetic diversity than non-Africans due to a dispersal event out of Africa that left non-Africans with a subset of African variation [27–29]. Further, African populations have significant substructure [30, 31] and deep patterns of demographic history [32] that lead to wide variation in observed diversity. These demographic processes differentially affect regions of the genome with different relative population sizes (e.g., the X chromosome and the mitochondria) [33]. Similarly, sex-biased demographic processes (e.g., different number of mating males and females, sex-biased admixture, or migration) can shape differences in diversity between regions like the sex chromosomes and the mitochondria whose inheritance patterns are governed by the sex of the individual [15, 34–36].

The X chromosome contains several distinct regions that have different evolutionary histories, and which operate under various combinations of the above processes. The sex chromosomes (X and Y) in mammals diverged from a pair of autosomes approximately 180–210 million years ago [37]. Over time, the X and Y evolved to have different structure and gene content, with the Y chromosome losing about 90% of its original genes [38, 39]. This differentiation has been theorized to be a result of a handful of inversion events on the Y [40–43] that lead to reduced recombination between the X and Y chromosomes. Homologous recombination does not occur along much of the length of the X and Y chromosome. However, they share two pseudoautosomal regions (PAR1 and PAR2). PAR1 extends ~2.7 Mb from the tip of the proximal arm of each sex chromosome and facilitates X-Y recombination [38, 43]. PAR2 extends 320 kb on the tip of the long arm of each sex chromosome, and evolved independently from PAR1 as a result of at least two X to Y duplication events [44, 45]. Recombination rate varies significantly across regions of the X chromosome due to X-Y recombination being constrained to the PARs; PAR1 recombination rate is ~20x the genome average [46] and PAR2 recombination rate is ~5x the genome average [47]. In addition to the two PARs, there is an X-transposed region (XTR) in humans that was duplicated from X to Y around 3 to 4 million years ago, after human-chimpanzee divergence [38, 48–50]. The XTR has undergone a series of inversions and deletions, but it maintains ~98% X-Y sequence identity [38, 51] and contains two genes with functional X-Y homologs [49].

The evolutionary history of the pseudoautosomal regions has been well studied [52, 53]. Variation has been shown to be better maintained in these regions, with processes such as sexually antagonistic selection (and other sex-specific selection) affecting diversity [52, 54]. Fine scale maps of sex-specific recombination differences between PAR1 and PAR2 have also recently shed light on processes shaping pseudoautosomal diversity [53]. Recombination alone is not sufficient in homogenizing genetic differences between the X and Y pseudoautosomal regions, and the mechanism by which these differences arise remains unclear [53]. Clearly, the human pseudoautosomal regions have been distinctly shaped by recombination and other evolutionary processes–what remains unclear is how both demographic and genomic processes affect different regions of the X and specifically how the impact of these processes varies when analyzing each of the X chromosome regions across different human populations.

Because of its unique structure, inheritance, and evolutionary history, the X chromosome serves as a unique model for studying how genetic and demographic forces act in different contexts to shape patterns of observed variation. For example, departures from neutral equilibrium expectations of X/A diversity have been used to study sex biases in processes such as migration, admixture, generation time, and reproductive success [15, 19–21, 24, 36, 55, 56]. While recombination between the X and Y pseudoautosomal regions has been studied comparatively, and the non-pseudoautosomal X has been studied relative to the autosomes [21, 53], we lack a complete picture of how all of the X chromosome regions behave relative to each other. In this study we expand on these previous analyses in two ways: (1) by separately considering all individual regions of the X chromosome relative to the autosomes and (2) by studying these regions in a large, global sample of humans (2,504 individuals from 26 different populations sequenced as part of the 1000 Genomes Project [57]) that have experienced a range of different demographic histories. From this data, we calculate measures of diversity, divergence, and linkage disequilibrium to investigate the extent to which linked selection, recombination, mutation rate, and demography shape relative patterns of variation across the human X chromosome. This design allows us to better understand the forces that shape genetic variation and gives a detailed look into the evolutionary biology of the X chromosome.

## Results

### Genetic variation is consistently elevated in the PARs and XTR across human populations

**Fig 1:**
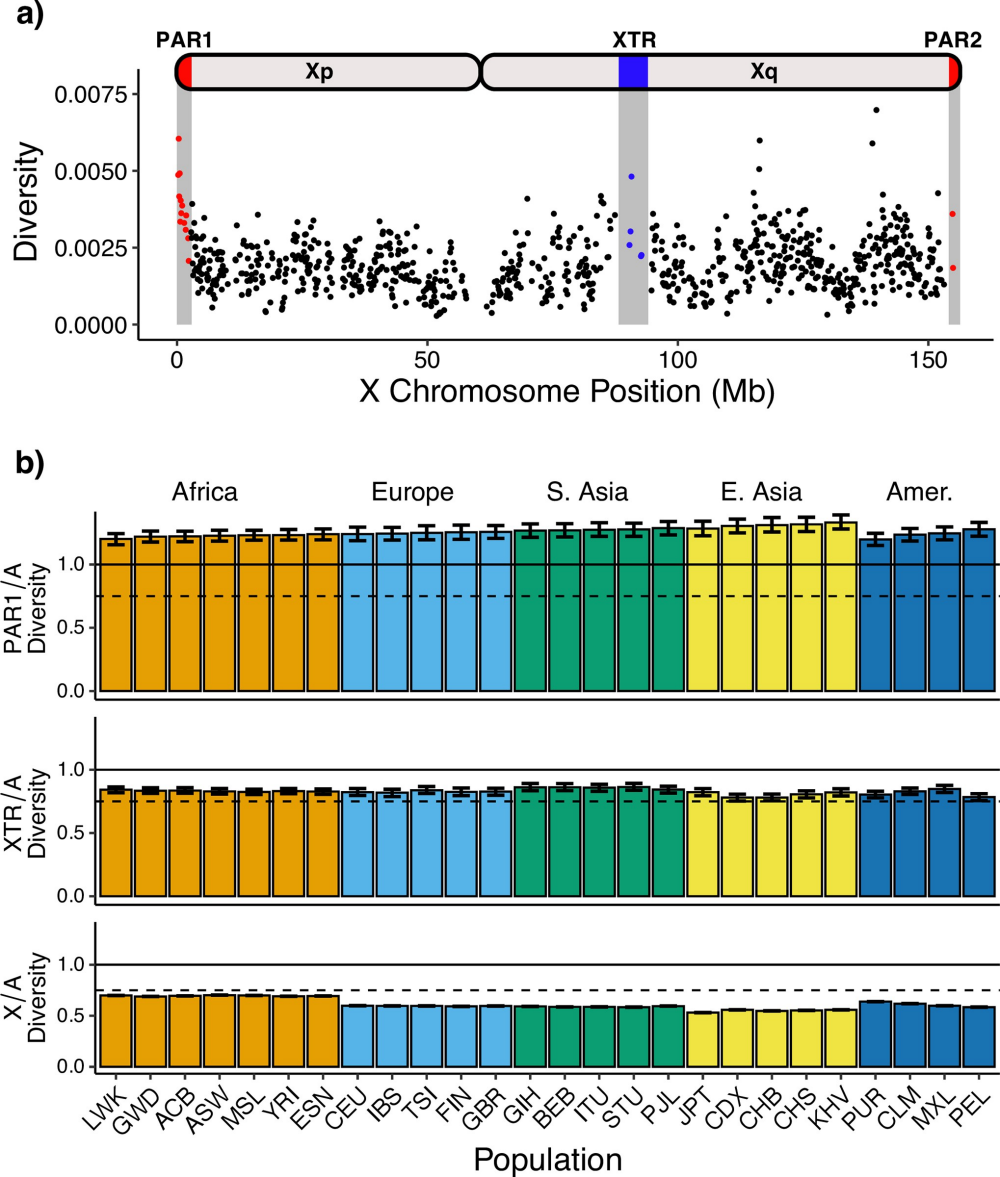
Genetic diversity across regions of the human X chromosome. a) Nucleotide diversity is calculated in non-overlapping 100kb windows across the X chromosome and corrected for mutation rate variation using hg19-canFam3 divergence. Red indicates the pseudoautosomal regions (PAR1, PAR2) and blue indicates the X-transposed region (XTR). Diversity is calculated using all 1000 Genomes phase 3 samples. b) Genetic diversity is calculated in each population between each region of the X chromosome—pseudoautosomal region 1 (PAR1), X-transposed region (XTR) and chromosome X—and the autosomes (chr8). Autosomal and X-linked diversity are corrected for mutation rate (hg19-canFam3 divergence). The solid line at 1.0 represents the null expectation of four PAR1 regions for every four autosomes. The dashed line at 0.75 represents the null expectation of three X chromosomes for every four autosomes. Error bars represent 95% bootstrapped confidence intervals using 1000 replicates. Populations are organized by superpopulations. Individual population abbreviations are labeled, and full names are available in S1 Table.

We measured nucleotide diversity in 26 human populations (S1 Table) from the 1000 Genomes Project [57]) and observed substantial variation across regions of the X chromosome (Fig 1A). Overall, we found that diversity is significantly higher in both PAR1 and XTR than chrX (which we define here as non-pseudoautosomal sequence on the X chromosome not in PAR1, PAR2, or XTR) in nearly all populations (S2 Table). We also observed higher diversity in PAR2 than chrX in all cases, but the difference was never significant. After filtering, PAR2 has approximately 15% as many variant sites as PAR1 and approximately 65 kb of callable sequence (S3 Table). Due to its size, unusual evolutionary history, and the small amount of data available after filtering, we report observational results for PAR2 but exclude it from interpretations.

### Ratios of PAR/A and chrX/A diversity exhibit opposite patterns across human populations

To further explore differences in diversity among regions on the X chromosome, we divided diversity values calculated in PAR1, XTR, and chrX by those from an autosome approximately the same size as the X, chr8 (referred to here as autosome or A). We do this for each of the 26 1000 genomes populations (Fig 1B). The nonPAR chrX/A values were below the null expectation of 0.75 (assuming equal sex ratios and 3 X chromosomes for every 4 autosomes). PAR1/A ratios were all greater than 1.0, and thus greater than expectations based on chromosome counts (i.e., two copies of chromosome 8 in all individuals, and two copies of PAR1, either on two X chromosomes in females or on the X and Y in males). We observed PAR1/A ratios around 1.25 within Africa, and gradually increasing PAR1/A ratios in populations outside of Africa. In contrast, chrX/A ratios decreased in populations outside of Africa. This pattern was recapitulated in admixed American populations, in which we observed that PAR1/A ratios increased with decreasing African ancestry proportions, while chrX/A ratios decreased with decreasing proportion of African ancestry. In order of decreasing African ancestry proportion, those populations are Puerto Rican (~28% African ancestry), Colombian (~7%), Mexican (~4–5%), and Peruvian (~2%) [58–62].

Surprisingly, our observations of XTR did not match our expectation that it would behave similarly to chrX because both are present in one copy in genetic males and two copies in genetic females. Across populations, XTR/A diversity was consistently greater than observed chrX/A diversity (Fig 1B) and variation in XTR/A ratios did not appear to correspond with demography.

### Substitution rate varies across regions of the human X chromosome

**Fig 2:**
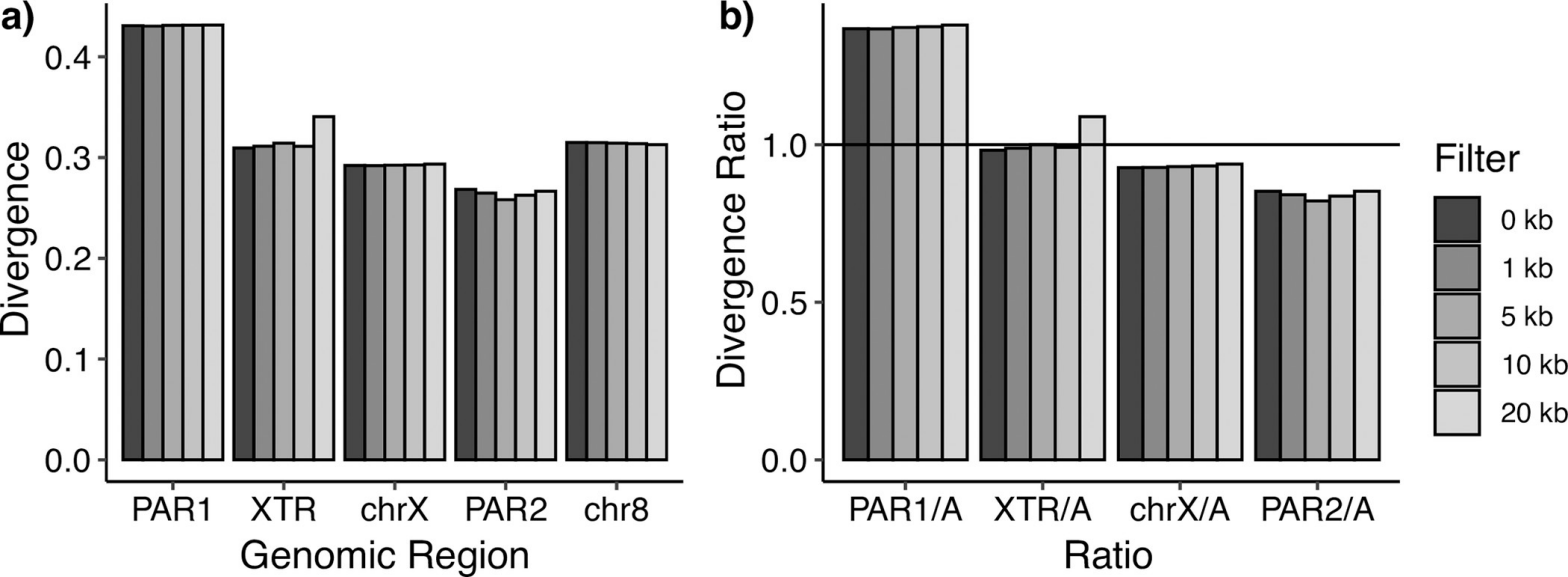
Divergence across the genome. Human-dog divergence (hg19-canFam3) a) across regions of the human X chromosome and chromosome 8; and b) between each region of the human X chromosome relative to chromosome 8. A solid horizontal line is placed at a divergence ratio of 1, which would imply an equal substitution rate between regions. Divergence is computed for all intergenic regions with no filter with distance from genes (0kb), or with a filter removing regions near genes (1kb, 5kb, 10kb or 20kb). The number of base pairs in each region is reported in S3 Table.

Mutation rate, which is known to vary across the genome [63–65], influences observed levels of genetic diversity. Under a neutral model of evolution, higher mutation rates result in more genetic variation and thus increased levels of diversity [66]. To explore regional variation in mutation, we used substitution rate (divergence) between the human and dog reference genomes as a proxy. In general, divergence did not increase with increasing distance from genes (Fig 2), though the XTR exhibited a slightly elevated substitution rate in the bin removing 20kb from both sides of genes and PAR2 substitution rate tended to fluctuate slightly over all bins (S3 Table).

While we did not find an association between human-dog divergence and distance from genes, we observed striking differences in substitution rates across the different regions of the X chromosome and chromosome 8 (Fig 2). PAR1 had the highest substitution rate (~1.3x that of chr8), while the substitution rate of the XTR was similar to that of chromosome 8. Both chrX and PAR2 had lower substitution rates than chromosome 8. For chrX, the difference was slight (0.93x that of chr8).

### Linkage disequilibrium in PAR1 and XTR is lower than elsewhere on the X chromosome

**Fig 3:**
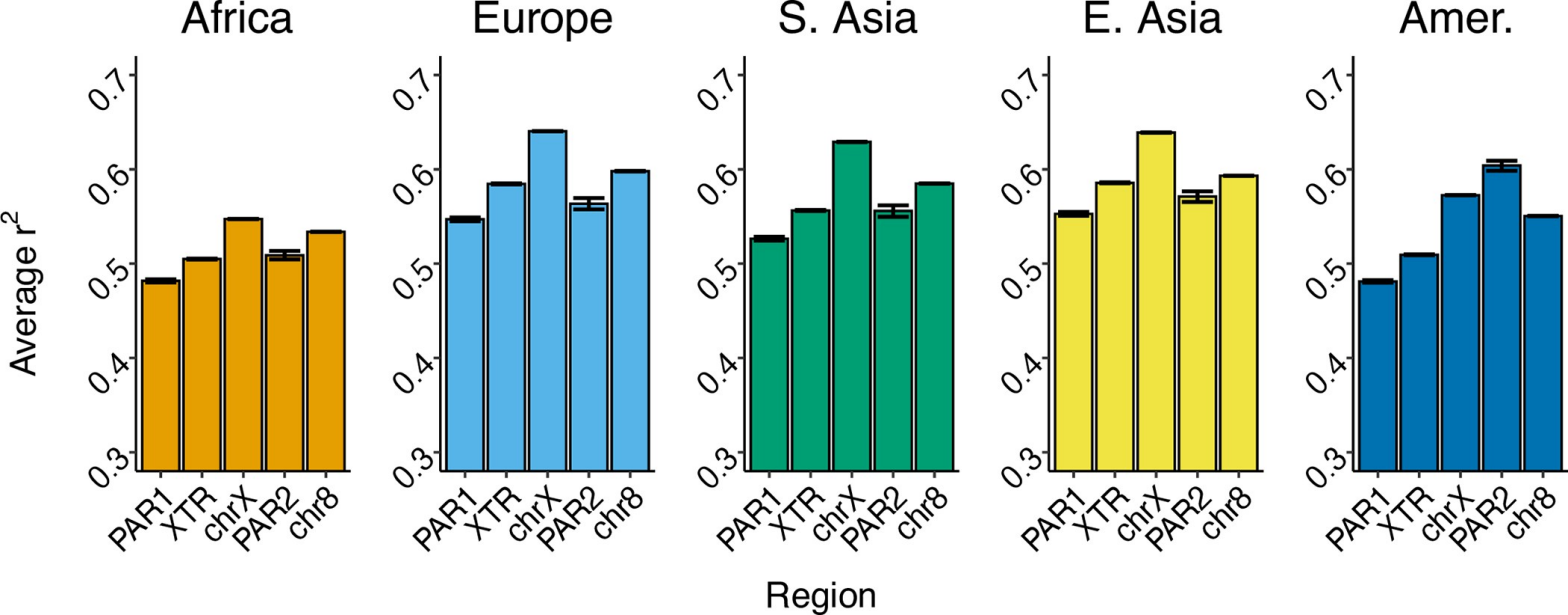
Average linkage disequilibrium across genomic regions. Linkage disequilibrium (LD) is calculated in each X chromosome region and chromosome 8 for each superpopulation. LD is calculated for each site in a given genomic region by averaging all pairwise *r*^*2*^ values +/- 300kb from that site. Average *r*^*2*^ values for each site are then used to calculate mean LD for a given region. Error bars represent 95% bootstrapped confidence intervals (1000 replicates).

We calculated average *r*^*2*^ across the X chromosome and chromosome 8 to characterize linkage disequilibrium (LD) as a proxy for recombination rate (see Methods). Consistent with our expectation that a higher recombination rate will break up linkage disequilibrium, we found that LD is lowest in PAR1 and highest in chrX, with chr8 exhibiting values slightly lower than chrX (Fig 3). However, the XTR exhibited intermediate *r*^*2*^ values that fell approximately halfway between PAR1 and chrX (22 of 26 populations, S1 Fig). Estimates for LD in PAR1 and the XTR varied slightly among populations within the same superpopulation (S1 Fig), but these trends are broadly consistent across all populations studied. Trends in LD between different populations rather than between different regions could potentially be driven by other factors, such as larger historical effective population size in African populations.

### Genetic diversity and linkage disequilibrium are negatively correlated on the X chromosome

To examine the relationship between genetic diversity and LD and to explore how this relationship is affected by filtering with distance from genes, we characterized the correlation between LD and diversity calculated in the same set of windows [67]. Estimates of genetic diversity should decrease with decreasing LD due to the increased effect of recombination breaking up linked selection. It follows that as distance from genes increases, the decreasing effect of linked selection should correspondingly weaken the relationship between LD and diversity. We found a significant negative correlation between LD and diversity (R^2^ = 0.127, P = 2.46x10^-31^; S2A Fig). We also observed that LD explains less variation in diversity when we filtered to include only sequences further from genes (R^2^ = 0.10, P = 8.03x10^-22^; S2B Fig).

### Regions of the X chromosome exhibit contrasting and population-specific patterns of linked selection

**Fig 4:**
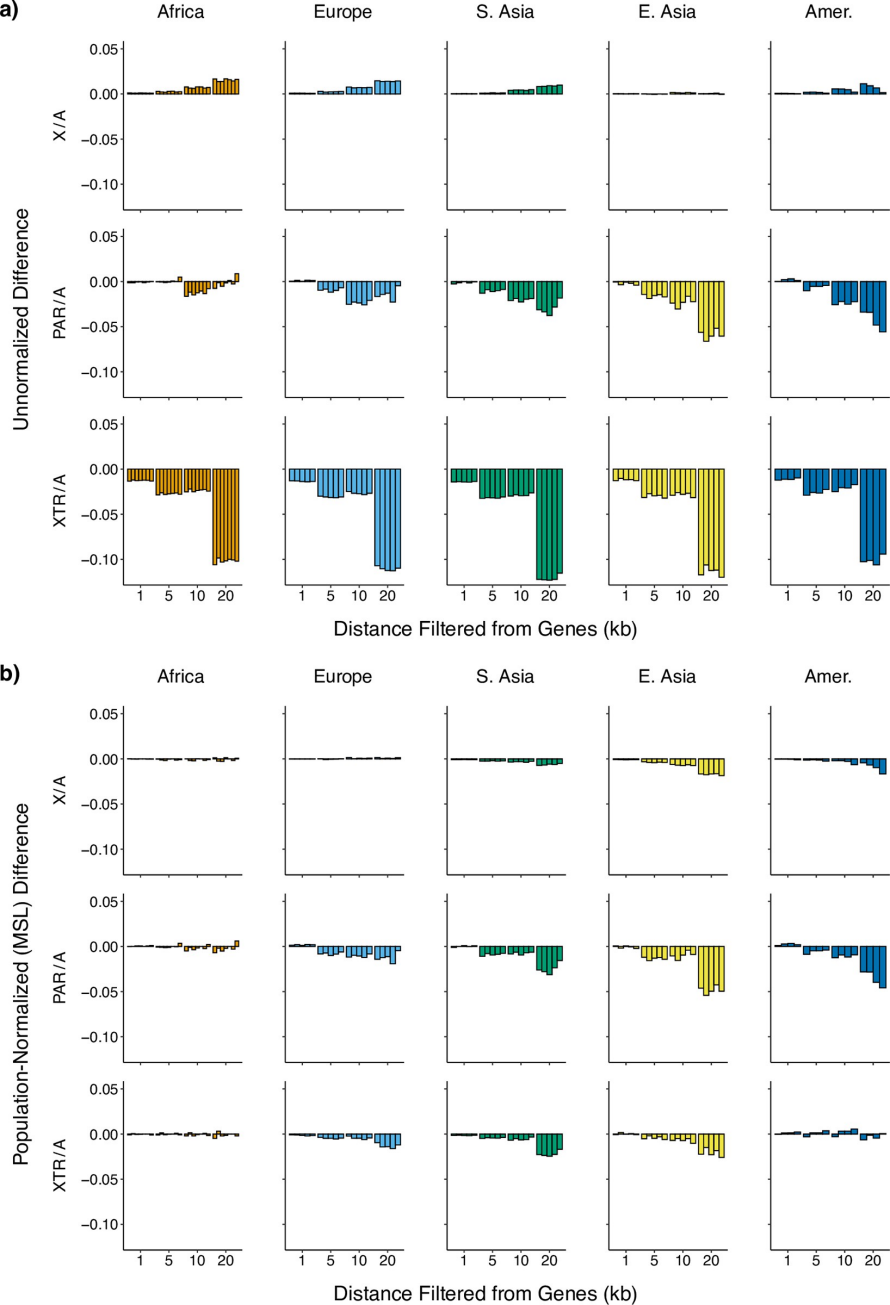
Ratio of X to autosomal diversity with increasing distance from genes across populations. a) Diversity ratios (corrected for hg19-canFam3 divergence) are reported between regions on the X—non-pseudoautosomal X (X), pseudoautosomal region 1 (PAR), and X-transposed region (XTR)—and the autosomes for 26 populations from the 1000 Genomes Project. Values are reported as the difference between using a filter for only genes and a filter removing for 1kb, 5kb, 10kb, and 20kb from genes, respectively. The order of populations is the same as reported in Fig 1B. b) These ratios are demography normalized by reporting each population relative to Mende in Sierra Leone (the population with the highest nucleotide diversity among all populations for most X-chromosomal regions).

We also explored how X/A, PAR/A, and XTR/A diversity ratios varied across 26 populations in Africa, Europe, South Asia, East Asia, and the Americas as we removed regions close to genes (Fig 4A). We calculated diversity in each population after removing genes and conserved sequences. We then iteratively removed regions of increasing size, starting from the region closest to genes and moving out to a designated threshold (1kb, 5kb, 10kb, 20kb). The measure we use is the difference between (a) diversity calculated after removing the flanking sequence from genes and (b) diversity calculated where we only remove genes. More efficient selection on the X chromosome should lead to patterns of increasing X/A diversity ratios moving away from genes due to more pronounced linked selection on the X chromosome [19–21]. Consistent with this prediction, we found that X/A diversity ratios increased as we used filters that removed longer regions of sequence close to genes in four of the five superpopulations (Fig 4A). In contrast, we surprisingly found that PAR/A and XTR/A diversity ratios decreased as we filtered out these same regions.

To account for the effect that demography may have on these patterns, we corrected the ratios for each of the above populations by dividing these ratios by those from the African population MSL (Mende in Sierra Leone)—the population in our dataset exhibiting the greatest diversity across most regions of the X chromosome (Fig 4B). If patterns of linked selection are consistent across populations, we expect the normalized differences to be equal to 0 across all regions with increasing distance from genes [21]. Overall, we found that while most populations had normalized X/A ratios that did not change with distance from genes, some non-African populations, particularly those from East Asia, displayed values that decreased with distance from genes (Fig 4B).

For PAR/A and XTR/A ratios, the “flattening” effect of normalizing to MSL was less pronounced (Fig 4B). Although there is no effect of distance from genes on the normalized ratio in African populations, the normalized PAR/A ratio still decreases in the other four non-African superpopulations. Similarly, normalized XTR/A ratios appear to decrease with distance from genes in non-African superpopulations.

To look at the effect of different outgroup populations on normalizing X/A ratios, we divided diversity ratios for the 26 populations by the populations with the highest diversity in each of the four remaining superpopulations (S3A–S3D Fig). When we normalized the 26 populations by the Tuscan population (TSI; the highest diversity population in Europe for most of the X-chromosomal regions), we found that X/A, PAR/A, and XTR/A ratios in the remaining European populations were unaffected by increasing distance from genes with PAR/A and XTR/A ratios tending both upwards and downwards across the other four superpopulations (S3A Fig). We repeated this normalization for each of the three remaining superpopulations (S3B–S3D Fig) and each one exhibited the same pattern described above: X/A, PAR/A, and XTR/A ratios were unaffected by distance from genes for the superpopulation used for the normalization, while PAR/A and XTR/A ratios varied among the other superpopulations depending on the population chosen as the denominator.

## Discussion

There are many processes that shape the landscape of genetic variation across the genome. Patterns of genetic variation across the X chromosome are especially complex because its unique structure and pattern of inheritance have the potential to interact with these processes in different ways. In this study, we examined 26 diverse human populations and found remarkable variation in genetic diversity on the X chromosome, both among populations and across different regions of the X chromosome itself. More specifically, we found that the landscape of genetic variation across the X chromosome was structured by mutation, recombination, and population history, which differentially affected major regions of the X chromosome—the PAR, XTR, and nonPAR—and led to substantial variation in genetic diversity across these regions.

### The X-transposed region has intermediate properties of both PAR1 and nonPAR

Of the X-chromosomal regions we studied, our results for the X-transposed region (XTR) were especially surprising because its properties were intermediate to both the pseudoautosomal regions and the nonPAR regions of the X chromosome. Though the XTR shares homology with the Y chromosome, we expected it to behave similarly to the nonPAR regions of the X chromosome because it underwent an inversion preventing recombination [51]. However, in our measures of diversity (Fig 1B) and recombination (Fig 3), the XTR exhibited values that were greater than we observed in nonPAR, but less than we observed in PAR1.

The unusual pattern of diversity within the XTR could be driven, in part, by technical artifacts. We recently showed that, due to homology, the X-transposed sequences between the X and the Y are similar enough to confound the mapping of raw sequencing reads [68]. This leads to lower mapping quality and sequencing depth, which in turn reduces the number of variants called [68]. As this mapping correction was not implemented in the 1000 Genomes dataset, our observations of higher genetic diversity in the XTR than the nonPAR in this study are still surprising, as they are likely underestimated, having been published before the problem and correction were described by Webster *et al*. [68].

Our observation of lower linkage disequilibrium (LD) in XTR, if LD serves as a good proxy for recombination in this case, is consistent with recombination in this region. This is unexpected because, despite its X-Y homology, the XTR experienced an inversion event that is proposed to have prevented further recombination from occurring [51]. Our results are consistent with research suggesting that there is evidence for unequal crossing over [69] in the XTR for a small portion of the population, leading some researchers to dub this region PAR3 [50]. Though it remains unlikely that the X-Y recombination in this region is extensive, the substantial difference between XTR and nonPAR that we observed in this study should motivate further molecular investigations of the XTR to better understand this behavior.

We propose that there are two additional explanations for the observed XTR/A diversity. First, is that the XTR has only recently started to diverge between the X and Y chromosomes, so may reflect a transition state between a fully recombining region with an autosomal effective population size (like the pseudoautosomal regions) and a region with a lower effective population size (like the nonPAR/nonXTR X chromosome regions) that does not typically recombine in genetic males. Thus, there could be a biological expectation that diversity on the XTR relative to autosomes should be intermediate to the PAR/A and X/A regions. Alternatively, building on our previous description of technical artifacts in this region [68] and in accordance with previous observations about X-transposed region diversity [70], it is possible that genetic diversity in the XTR is actually lower than measured, but that mis-mapping of Y-linked reads results in a technical error that increases measured diversity in this region. Many genome-wide studies remove the PARs when analyzing the X chromosome, but for the reasons above, we suggest that it is equally important to remove the XTR and consider it separately. Long-read genomic data, which can be used to span gaps and highly-repetitive regions of the X chromosome, could also provide additional capacity to assess the variability in this region [71]. The intermediate behavior of the XTR merits further study to dissect the relative contributions of these technical and biological effects.

### Recombination influences mutation rate across X-chromosomal regions

While some past studies have generally concluded that genetic divergence is not associated with recombination hotspots across the autosomes [72], other work has shown that double-strand-break repair can be mutagenic in species like *S*. *cerevisiae* [73]. Additionally, recent analyses in large human cohorts have revealed an association between *de novo* mutations and recombination hotspots [74]. Here, we have observed a correlation between LD (our proxy for recombination rate) and substitution rate (our proxy for mutation rate) on the X chromosome when considering each of our X-chromosomal regions of interest. Our substitution rate observations (Fig 2) are consistent with mutation rate being higher in PAR1 and XTR than the nonPAR regions of the X chromosome. Similarly, our linkage disequilibrium estimates (Fig 3) are consistent with higher recombination in PAR1 and XTR than nonPAR. PAR1 has been previously observed to have increased substitution rate relative to autosomes [75]; a result confirmed here. This phenomenon supports the conclusion that recombination rate is positively correlated with mutation rate. Further, this pattern is replicated in 22 of 26 populations (S1 Fig), consistent with it having a more general biological explanation, rather than a demographic one.

Additionally, it has been argued that the correlation between recombination rate and genetic diversity in the human PAR1 is driven specifically by the relationship between recombination rate and divergence [76]. Here our observations expand this observation to the other regions of the X chromosome—specifically the XTR—and suggest a complex interplay between recombination and mutation rate in shaping genetic diversity across the regions of the X chromosome.

### The X chromosome exhibits patterns of linked selection that differ among populations

The ratio of X chromosome to autosome diversity has long been of interest in exploring aspects of population history, particularly those that are sex-biased [15, 19, 21, 56, 77]. Analyzing these ratios in the same way across 26 human populations gives us an unprecedented look at how this measure changes both across a variety of demographic histories and across different regions of the X chromosome.

When considering just the nonPAR X, we observe a pattern largely in line with previous studies: the highest ratios are in African populations (Fig 1B). As population bottlenecks disproportionately affect the X chromosome because of its smaller effective population size [33], lower ratios outside of Africa were likely the result of a bottleneck in the population ancestral to all non-African groups when it was migrating out of Africa [28]. Other work has shown that strong male biases during this migration might have also decreased these ratios [78, 79]. Interestingly, when we organized admixed populations from the Americas based on the amount of African ancestry they contain, we recapitulated the same pattern: we observed decreasing X/A ratios with decreasing African ancestry. Thus, while there is clearly variation among individual populations, the migration out of Africa by some groups is by far the most dominant force shaping X/A ratios in humans.

In contrast to the nonPAR X, when we used XTR/A or PAR1/A ratios, we observed very different patterns (Fig 1B). Both ratios were significantly higher than expected, with XTR/A ratios greater than 0.75 and PAR1/A ratios greater than 1.0 in all populations. Moreover, for PAR1/A ratios, we observe an inverse demographic pattern to what we observed for X/A, with PAR1/A ratios increasing out of Africa and admixed American populations exhibiting increasing values with decreasing African ancestry. It is critical to note that the only difference among these analyses is the region being studied: PAR1, XTR, and the nonPAR X display these contrasting patterns within the same populations and under the same demographic histories. For the XTR, ratios higher than both the nonPAR X and a null expectation of 0.75 could be consistent with some recombination in this region, as discussed above, but it’s unclear why differences among populations don’t scale with those observed in nonPAR X and PAR1. Mutagenic recombination might explain the higher-than-expected PAR1 values overall, but it does not immediately explain the apparent increase in PAR1/A ratios out of Africa.

There are a few possibilities to explain this trend of increasing PAR1/A ratios. We observe that the interaction of differences in recombination (e.g., the high recombination rate difference between PAR1 and nonPAR X) and population history over human evolution (e.g., the out of Africa bottleneck and subsequent population expansion) could potentially offer one explanation for both the higher-than-expected PAR1/A ratios and the gradual increase in these ratios in non-African populations. Another possibility is the effect of balancing selection, which could maintain greater genetic diversity in PAR1 relative to the autosomes if it were acting disproportionately in this region [80, 81]. Another theoretical expectation for higher-than-expected diversity in the PARs is the effect of sexually-antagonistic selection maintaining variation in PAR1 and PAR2 more easily than the autosomes [54]. However, some recent work has argued that the effect of sexually-antagonistic selection may not fully fit with observed data [53].

When considering the effect of linked selection, we saw that nonPAR X/A ratios increase after filtering sequence close to genes which is consistent with the hypothesis that the X chromosome experiences more efficient diversity-reducing selection (i.e., hitchhiking and background selection) than the autosomes, due in part to it being found in only one copy in most genetic males [19, 21]. In contrast, we observed decreasing PAR/A and XTR/A ratios with distance from genes (Fig 4A) which could be consistent with multiple processes including less efficient selection and a higher density of elements under strong selection in these regions than the autosomes. Because different chromosomes vary in the landscape of their genetic variation [82, 83] due to unique distributions of structural variation, genes and conserved elements, and GC content, it’s possible that our results could have been affected by our choice of autosome (chromosome 8). However, we are unaware of evidence of large-scale differences in diversity among autosomes and expect our conclusions to hold across choice of denominator.

In order to learn more about the differences in the X/A, PAR/A, and XTR/A ratios across the populations we studied (Fig 1B), we considered relative ratios between sets of two populations (“normalized ratios”; Fig 4B). Previously, Arbiza et al (2014) plotted these normalized ratios as a function of distance from genes to separate the effects of demography and selection [21]. If normalized ratios don’t change with distance from genes, it implies that demography drives any observed differences in X/A diversity ratios among populations. However, if these ratios do change with increasing distance from genes, it suggests that population differences in patterns of selection can be shaping X/A diversity ratios as well.

Arbiza et al. (2014) found that, for two populations (from EUR and EAS), this normalization resulted in roughly equal X/A ratios near and far from genes, leading authors to conclude that the general relationship of selection between the X and autosomes was similar across human populations [21]. We replicated this normalization for PAR/A, XTR/A, and X/A ratios (using Mende in Sierra Leone, MSL, as our denominator for all populations) and observed the same result of Arbiza et al. (2014): no increase in X/A ratios with increasing distance from genes in African and European populations (Fig 4B). However, when we normalized using populations with vastly different demographic histories, we found some notable differences in X/A ratios which suggest that selective forces vary across human populations. First, PAR/A and XTR/A ratios always increase or decrease in populations outside of the superpopulation used for the normalization (with the sign of the effect also depending on the population used). Second, each ratio is unaffected by distance from genes when it is normalized within its own superpopulation. While there may be common effects of selection on the X chromosome in some populations, it is likely that different regions are under different selective pressures across different global populations.

Overall, our work builds on a growing picture that shows if we are to fully understand genomic variation and human evolutionary history, we need to look at a diversity of populations [84]. While normalization provides a simple, straightforward picture when considering two human superpopulations [21], the inclusion of additional populations demonstrates that this picture is far more complex and requires more nuanced interpretations. Many interpretations in population genetic studies depend on the choice of the populations that are being compared [85]. When making genomic claims, we must carefully consider the context of the populations that we are comparing. Further, analyses that include multiple genomic regions can shed light on how evolution shapes the genome as a whole. Without studying a diverse set of individuals from around the world we would not have been able to differentiate phenomena that seem to be shared across humans (e.g., biology of the XTR) versus those that vary among groups (e.g., patterns of linked selection on the X). Thus, the X chromosome is a uniquely important region for teasing apart both global and population-specific evolutionary processes.

## Methods

### Human DNA variation data

We obtained human genetic variant data in the form of VCF files from Phase 3 of The 1000 Genomes Project mapped to the reference genome hg19 [57]. We analyzed data from the X chromosome (chrX; ~155 Mb long) and chromosome 8 (chr8; ~146 Mb long), an autosome approximately the same length as the X chromosome, in 26 different populations from 5 major geographical regions (broadly, Africa, Europe, South Asia, East Asia, and the Americas; S1 Table). Throughout this paper, we use “superpopulation” to refer to the grouping of all individuals within a major geographical region (e.g., the superpopulation “Africa” refers to samples from all populations in Africa) and “population” to refer to one of the local populations (n = 26). We used the strict mask provided by the 1000 Genomes Project (*20141020*.*strict_mask*.*whole_genome*.*bed*) to assess callability and determine the number of monomorphic (i.e., invariant) sites in each region.

### Filtering regions of the genome

We used the UCSC Table Browser [86] to obtain coordinates for genomic elements that may be affected directly by selection or are difficult to align. We obtained coordinates for whole genes (transcription start to transcription end), centromeres, telomeres, CpG islands, and simple repeats. To curate a comprehensive and conservative list of whole genes we intersected records from the RefSeq genes track, the GENCODE genes track, and the UCSC genes track. We created iterations of this record with 0kb, 1kb, 5kb, 10kb, 20kb, 50kb, and 100kb of flanking sequence upstream and downstream of each gene additionally removed. For our main analyses, we used a filter that excluded 10kb flanking whole genes to better control for linked selection. We chose to remove sequences within 10kb of genes because removing greater distance from genes resulted in filtering much of the sequence from our regions of interest on the X chromosome (S3 Table). We processed all filter coordinates using bedtools [87].

### Divergence

To account for mutation rate variation, we corrected our diversity estimates in each region using pairwise divergence values between human and dog (hg19-canFam3) reference genomes. We used hg19-canFam3 divergence because substitution rates with more recently diverged primate species (rhesus macaque and marmoset) tended to correspond closely with the human PAR1 whereas in the dog comparison, variation in the substitution rate appeared to be independent of the human PAR1 boundary (S4 Fig). We obtained substitution rate estimates for each filter and window type by applying the Estimate Substitution Rate tool to sequence alignments from the Galaxy Toolbox [88] and correcting these results using the Jukes-Cantor 1969 model [89].

In addition to using these substitution rate estimates to account for variation in mutation rate, we explored how hg19-canFam3 divergence estimates within PAR1, PAR2, XTR, chrX, and chr8 change as we filter with increasing distance from genes (0kb, 1kb, 5kb, 10kb, 20kb). We additionally calculated divergence ratios between each of the X chromosome regions relative to chromosome 8 (Fig 2).

### Diversity calculations

We estimated uncorrected and unnormalized genetic diversity as the average number of pairwise differences per site (π) among sequences in each population. We used allele frequencies of single nucleotide polymorphisms to calculate diversity for each variant site:

π=1−∑i(ni2)(n2) (1)

where *n*_*i*_ is the allele count of allele *i* in a sample and *n* is the sum of *n*_*i*_ [90]. We calculated diversity across chrX and chr8 for each of the 26 1000 Genomes populations (S1 Table) in 1) non-overlapping 100 kilobase (kb) windows partitioned across each analyzed chromosome, and 2) distinct regions across the X chromosome: the pseudoautosomal regions located at the tips (PAR1 and PAR2), the X-transposed region located on the long arm (XTR), and the remaining regions (referred to simply as chrX; see Fig 1A). We obtained coordinates for PAR1 and PAR2 from build hg19 of the human genome and coordinates of XTR from Ross *et al*. [38]. See S4 Table for coordinates. In PAR1 and PAR2, variant calls in males were diploid and we calculated diversity using diploid calls across all males and females for each population of interest. For chromosome 8, we performed all calculations across the entire chromosome rather than calculating diversity in sliding windows. This bypasses any issues that might arise from structural variation on chromosome 8 that would affect window to window comparisons between it and the X chromosome [83, 91].

In each window/region we corrected for differences in mutation rate by dividing the window by the corresponding calculation of substitution rate in that window/region. For several of these windows on the long arm of the X chromosome, the divergence-corrected values are elevated as a result of high variability in the hg19-canFam3 substitution rates. We chose to include these windows because previous analyses of the X chromosome regions showed little effect of correcting for these high-diversity values on the results [70].

After correcting for divergence in each of the 100kb windows, we used permutation tests to compare mean diversity among the X chromosome regions for each of the 26 1000 Genomes populations. We divided chrX into 100kb non-overlapping windows and we permuted these windows 10,000 times to test the significance of the difference between diversity corrected for divergence in each X chromosome region (PAR1, XTR, PAR2) and the rest of chrX (S2 Table).

### Diversity ratios between the X and autosomes

To explore variation across the X chromosome regions (PAR1, XTR, chrX), we calculated the ratio of diversity corrected for divergence in each region relative to diversity corrected for divergence on chr8. We did this for each human population. We calculated 95% bootstrapped confidence intervals for these corrected diversity ratios (1000 replicates, resampling the values of diversity at each site in the region).

### Normalizing diversity for human demography

To explore the role that demography plays across these regions, we normalized diversity on the X and autosome, both corrected for divergence, by dividing by the population with the highest level of estimated diversity (in this case Mende in Sierra Leone; MSL). Thus, we have estimates of normalized diversity for 25 of the 1000 Genomes populations.

### Effects of linked selection on unnormalized and normalized diversity

To explore the effects that linked selection has on diversity, we analyzed sequence diversity with increasing distance from genes (0kb, 1kb, 5kb, 10kb, 20kb). To visualize the effects of removing potentially linked sequences, we plotted the difference in diversity between each filter that removed flanking regions from genes (1kb, 5kb, 10kb, and 20kb) with the measurement of diversity that only excluded genes and no flanking sequence (0kb). We did this both for unnormalized diversity and for measurements of diversity normalized to MSL (Fig 4B) as well as TSI, PJL, KHV, and PUR (S3A–S3D Fig).

### Linkage disequilibrium

We used linkage disequilibrium as a proxy to explore recombination rate variation across the X chromosome and chromosome 8. We first applied the same filters discussed above, removing 10kb of sequence flanking genes. We then calculated average *r*^*2*^ in 100 kb windows across each chromosome as well as within the X chromosome regions and all of chromosome 8. We did this separately for each superpopulation (Fig 3) and for each of the 26 1000 Genomes populations (S1 Fig). We considered each site individually and averaged all pairwise *r*^*2*^ values (calculated with Plink [92]) between that site and all other sites within 300kb in either direction. We then took the mean of each site’s average *r*^*2*^ values within each 100kb window and within each of our genomic regions (the X chromosome regions and chromosome 8). We estimated 95% bootstrapped confidence intervals by resampling 1000 times in each region of interest. To explore the relationship between LD and diversity, we used a linear regression analysis to compare the average *r*^*2*^ values and diversity values calculated in 100kb windows across the X chromosome (S2 Fig). All analyses were performed using the workflow manager, Snakemake [93].

## Supporting information

S1 Fig Average linkage disequilibrium across genomic regions. Linkage disequilibrium (LD) is calculated in each X chromosome region and for chromosome 8 for each 1000 Genomes Population. LD is calculated for each site in a given genomic region by averaging all pairwise *r*^*2*^ values +/- 300kb from that site. Average *r*^*2*^ values for each site are then used to calculate mean LD for a given region. Error bars represent 95% bootstrapped confidence intervals (1000 replicates with replacement).
(PDF)

S2 Fig Linkage disequilibrium and nucleotide diversity across the X chromosome. Average linkage disequilibrium was calculated in 100kb windows and plotted against corresponding average nucleotide diversity in 100kb windows (corrected for mutation rate with hg19-canFam3 divergence). This was done for a) diversity calculated by only filtering for genes and b) diversity calculated by filtering for genes +/- 10 kb flanking regions. *R*^*2*^ values for the negative correlation are reported on each plot.
(PDF)

S3 Fig Demography corrected ratios of X to autosomal diversity with increasing distance from genes across populations. Diversity ratios between regions on the X chromosome—non-pseudoautosomal X (X), pseudoautosomal region 1 (PAR), and X-transposed region (XTR)—and autosomes for 25 1000 genomes populations. Values are reported as the difference between using a filter for only genes and a filter including 1kb, 5kb, 10kb, and 20kb of sequences flanking genes. These ratios are demography normalized by reporting each population relative to a) Toscani in Italia; b) Punjabi from Lahore, Pakistan; c) Kinh in Ho Chi Minh City, Vietnam; and d) Puerto Ricans from Puerto Rico. The order of populations is the same as reported in Fig 1B (less the corresponding population used for the correction).
(PDF)

S4 Fig X chromosome substitution rates between human (hg19) and various outgroups. Substitution rates calculated in 100kb sliding windows and corrected using the Jukes-Cantor 1969 model [89] across the human X chromosome between the human reference (hg19) and *Rhesus macaque* (rheMac2; top), *Callithrix jacchus* (calJac3; middle), and *Canis lupus familiaris* (canFam3; bottom). Red indicates the pseudoautosomal regions (PAR1, PAR2) and blue indicates the X-transposed region (XTR).
(PDF)

S1 Table 1000 Genomes populations used in analyses. The number of male and female samples and population code used for each of the 26 1000 Genomes Project populations organized by superpopulation (African, Admixed American, East Asian, European, and South Asian).
(CSV)

S2 Table X chromosome diversity across populations. Nucleotide diversity was calculated for each 1000 Genomes population and normalized for mutation rate using canFam3-hg19 divergence. P values are calculated using a permutation method with 10,000 replicates for the difference between a region (PAR1, XTR, or PAR2) and nonPAR. P-values here are not multiple-test corrected.
(CSV)

S3 Table Filters with increasing distance from genes. The amount of data remaining for each filter was calculated with increasing distance from genes (0kb, 1kb, 5kb. 10kb, 20kb, 50kb, and 100kb). Callable sites, variants, uncorrected diversity measures, and diversity corrected to canFam3 divergence are reported for each X chromosome region (PAR1, chrX, XTR, PAR2), the Y chromosome, and chromosome 8.
(CSV)

S4 Table Gene density across the X chromosome and chromosome 8. Gene length and the number of genes are reported for each region across the X chromosome (PAR1, PAR2, chrX, XTR) and chromosome 8. (1) nonPAR is calculated as the remaining regions after removing PAR1, PAR2 and XTR. Thus, it is split up into two non-contiguous regions. (2) PAR1 and PAR2 coordinates come from the hg19 region definitions. (3) XTR is defined between 88 and 93 Mb [38]. It consists of two homologous blocks within this region and between the Y chromosome. We use these coordinates to be as conservative as possible.
(CSV)
