# Supplementary figures and images for: Genomic and demographic processes differentially influence genetic variation across the human X chromosome
Source: PLoS One. 2023 Nov 1;18(11):e0287609. doi: 10.1371/journal.pone.0287609 (PMC10619814; doi:10.1371/journal.pone.0287609)

Average  $r^2$

Africa

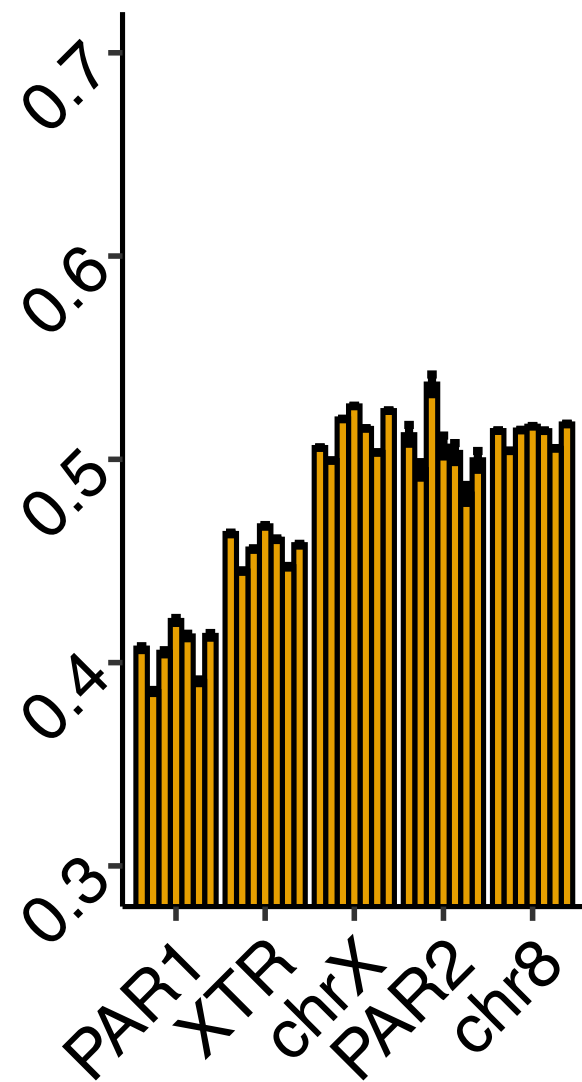

Europe

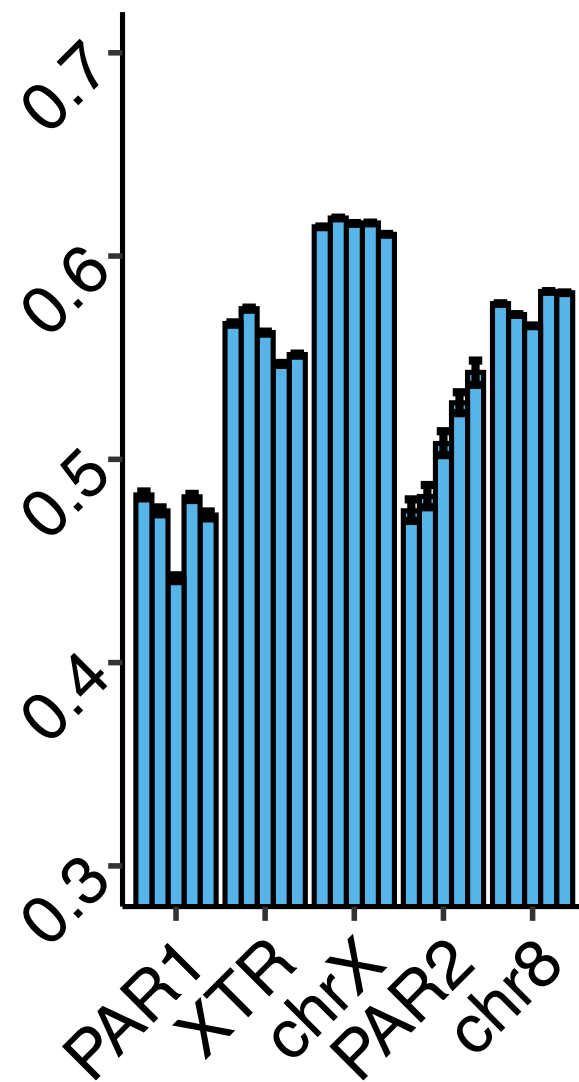

S. Asia

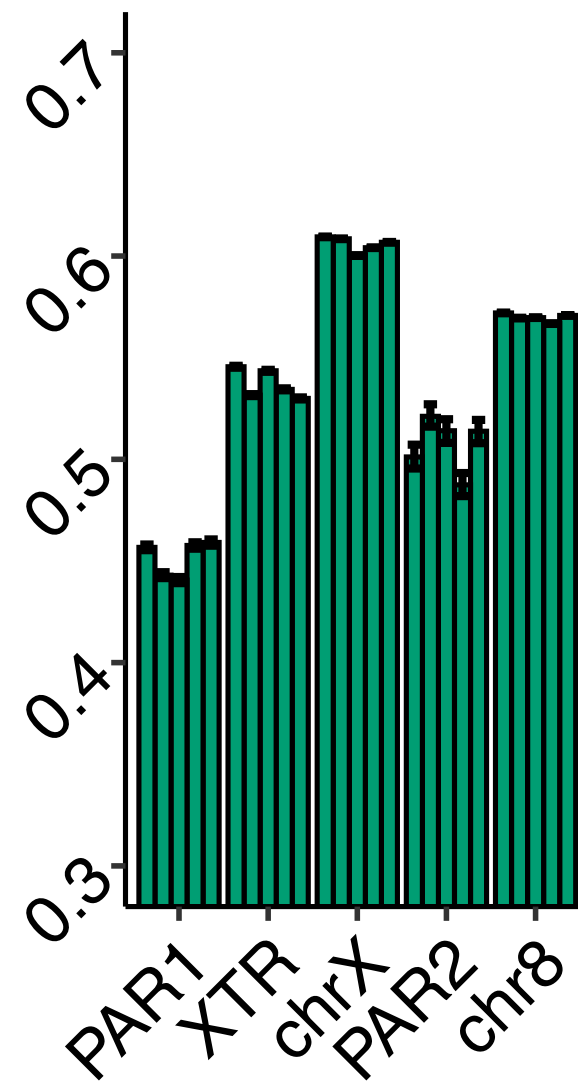

E. Asia

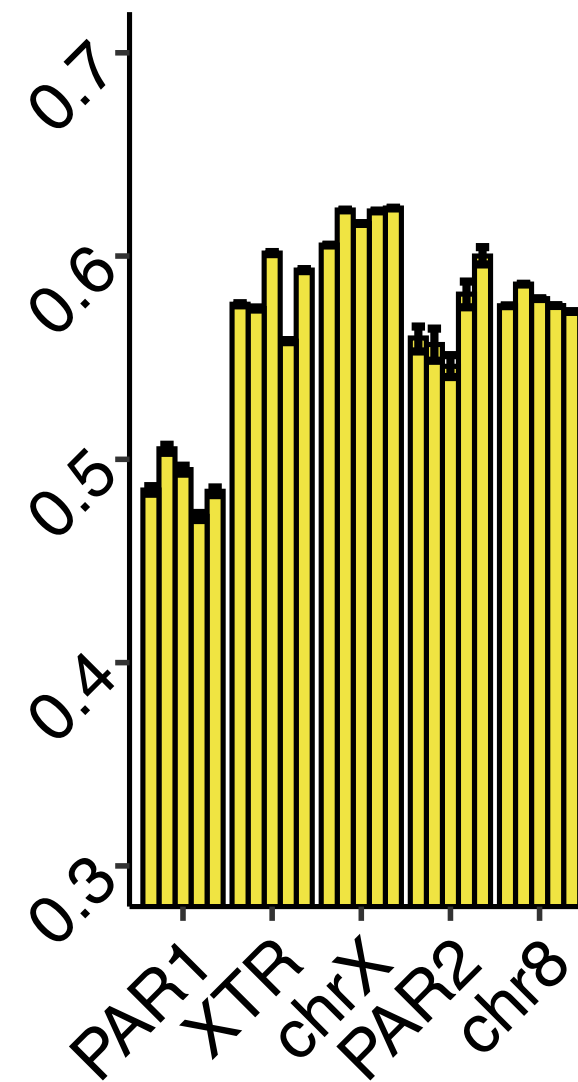

Amer.

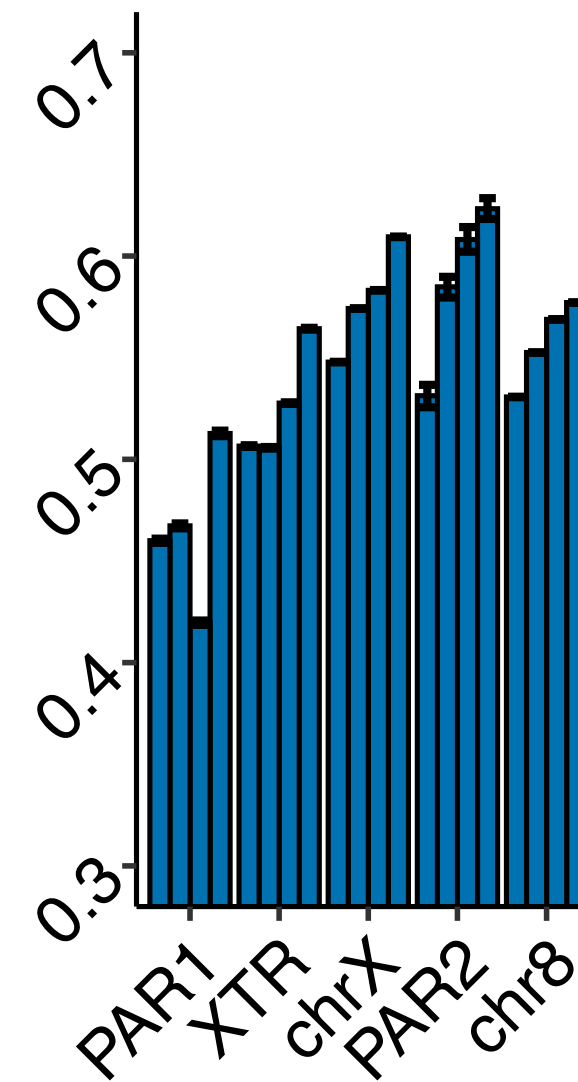

Region

Supplement: S1 Fig — Linkage disequilibrium (LD) is calculated in each X chromosome region and for chromosome 8 for each 1000 Genomes Population. LD is calculated for each site in a given genomic region by averaging all pairwise r2 values +/- 300kb from that site. Average r2 values for each site are then used to calculate mean LD for a given region. Error bars represent 95% bootstrapped confidence intervals (1000 replicates with replacement). (PDF) [file pone.0287609.s001.pdf]

a)

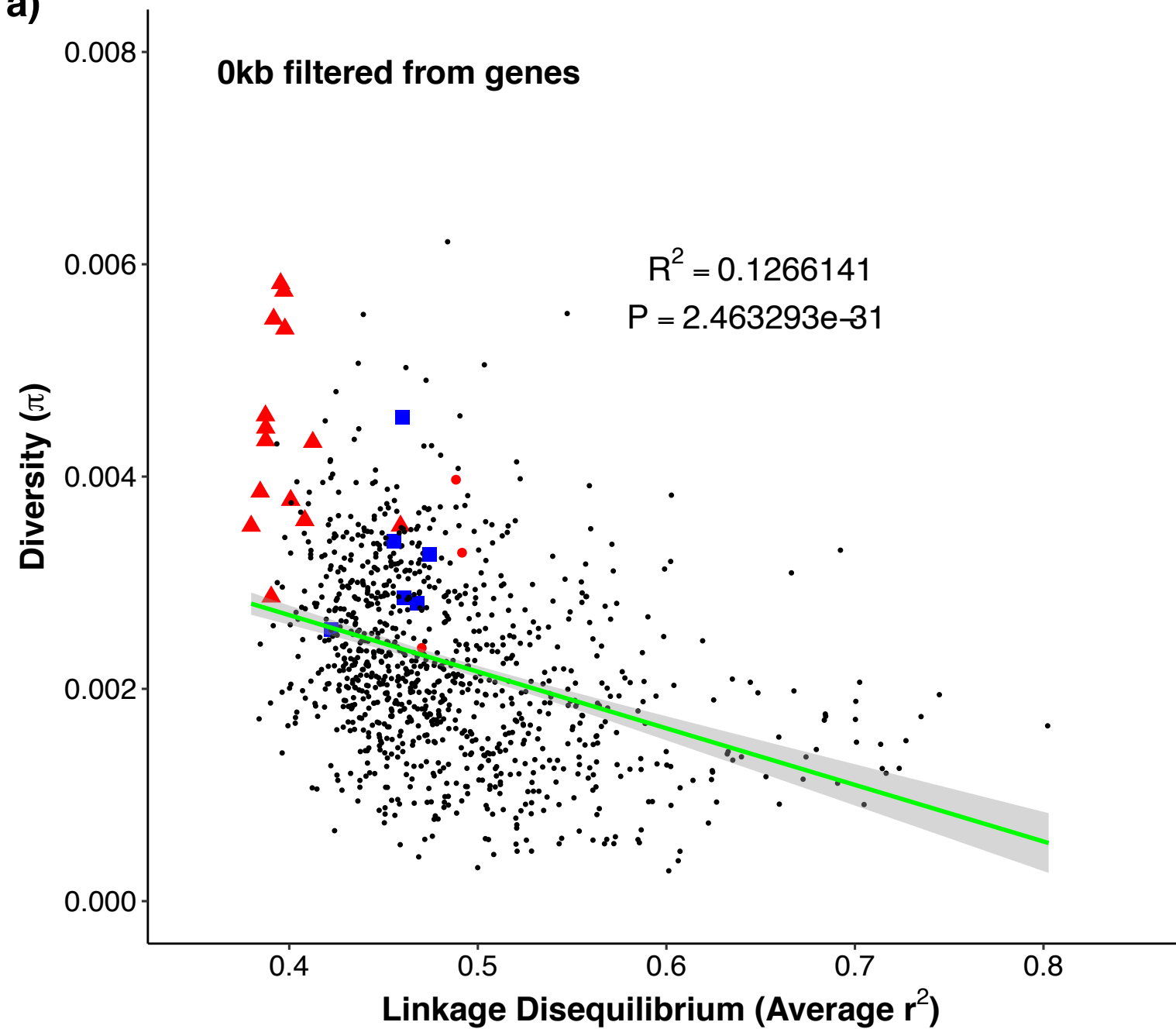

b)

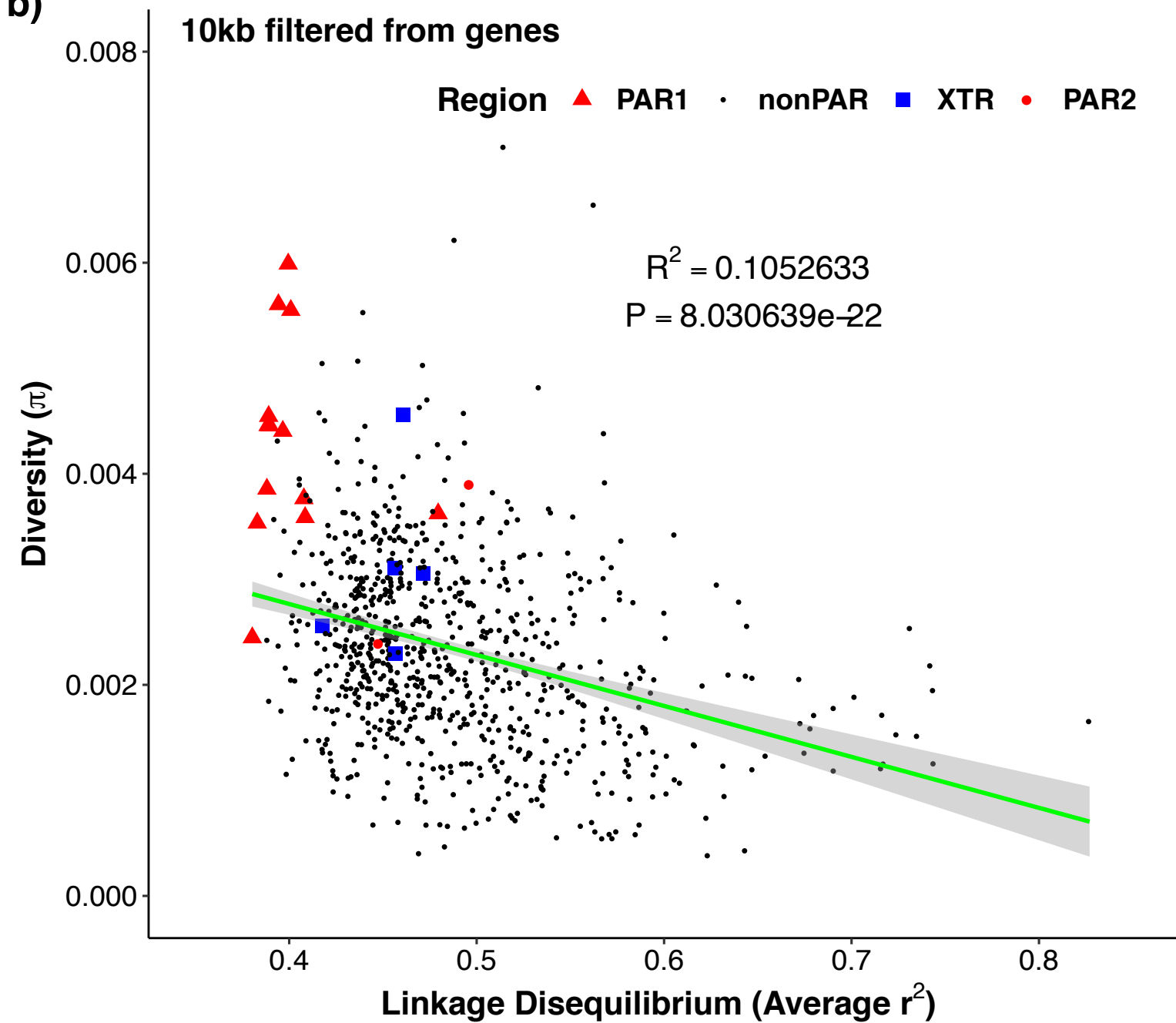

Supplement: S2 Fig — Average linkage disequilibrium was calculated in 100kb windows and plotted against corresponding average nucleotide diversity in 100kb windows (corrected for mutation rate with hg19-canFam3 divergence). This was done for a) diversity calculated by only filtering for genes and b) diversity calculated by filtering for genes +/- 10 kb flanking regions. R2 values for the negative correlation are reported on each plot. (PDF) [file pone.0287609.s002.pdf]

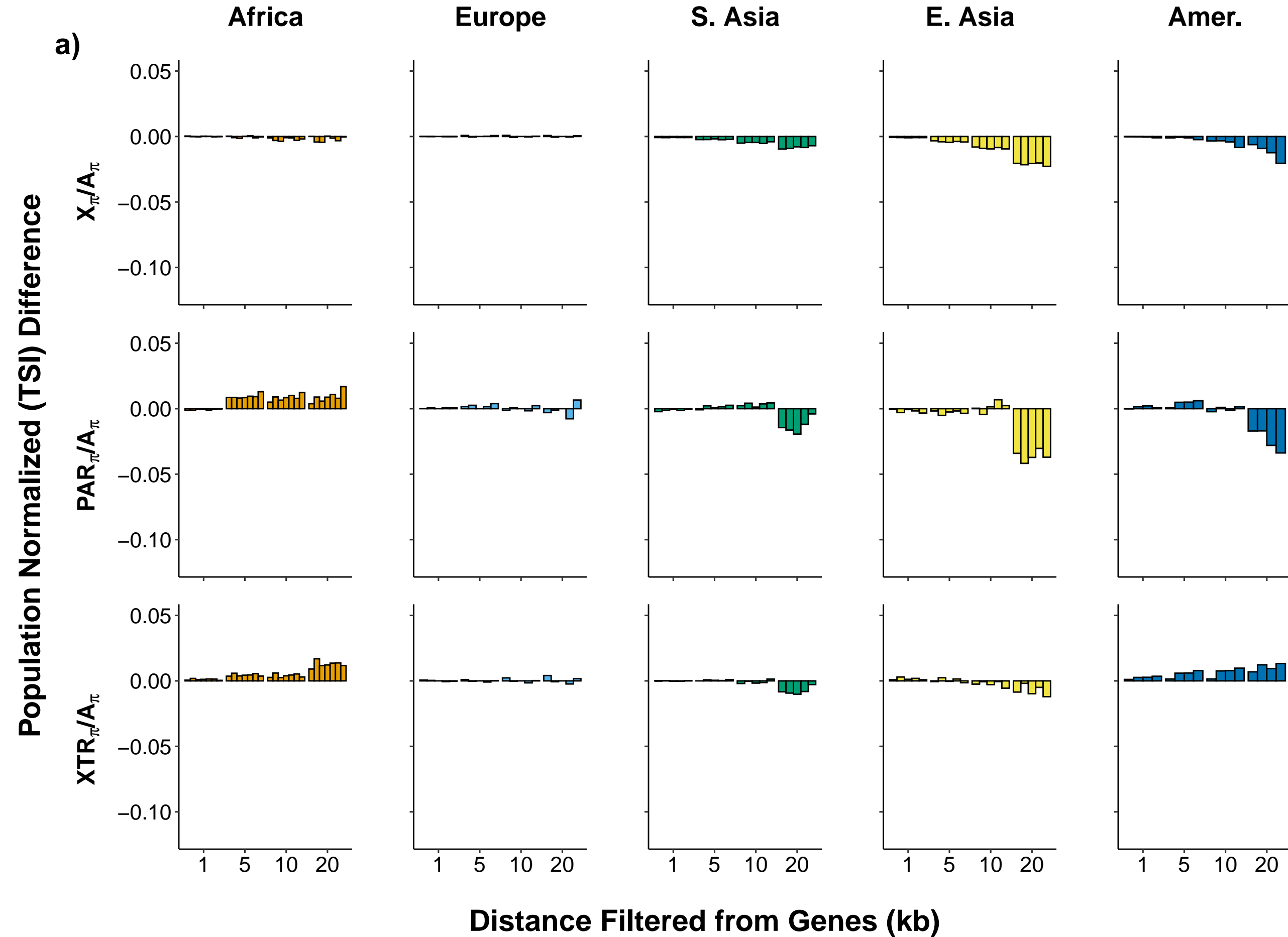

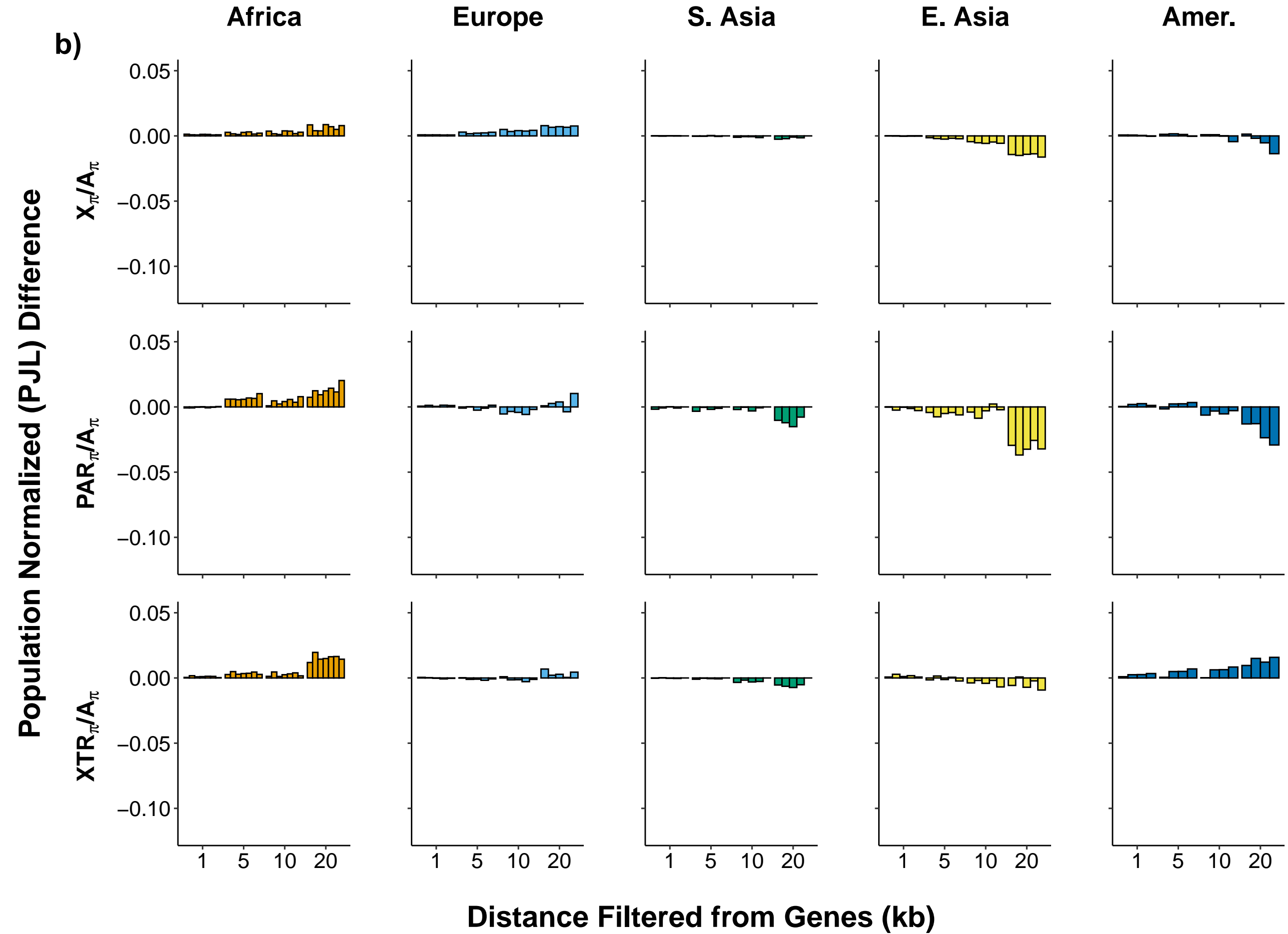

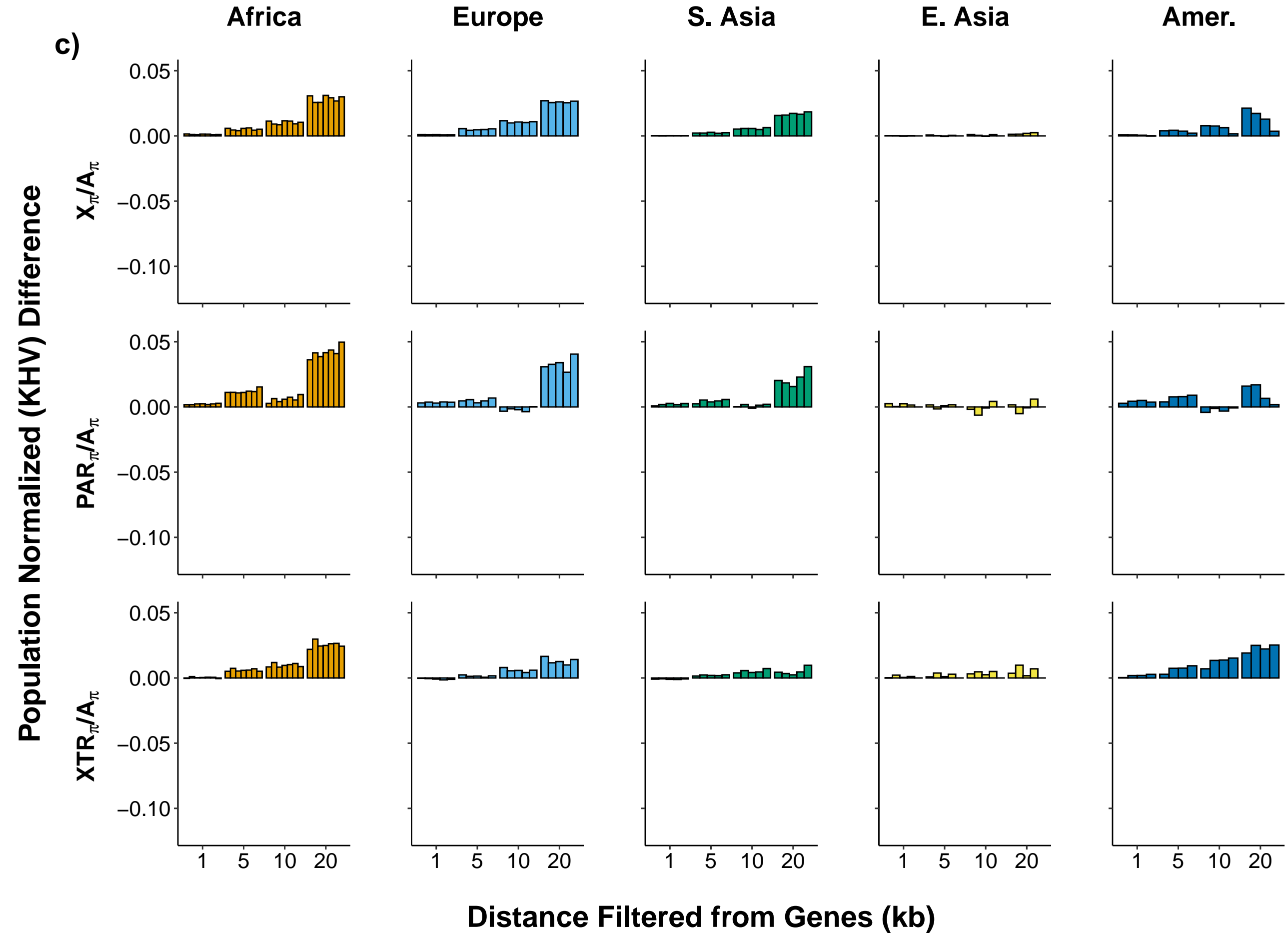

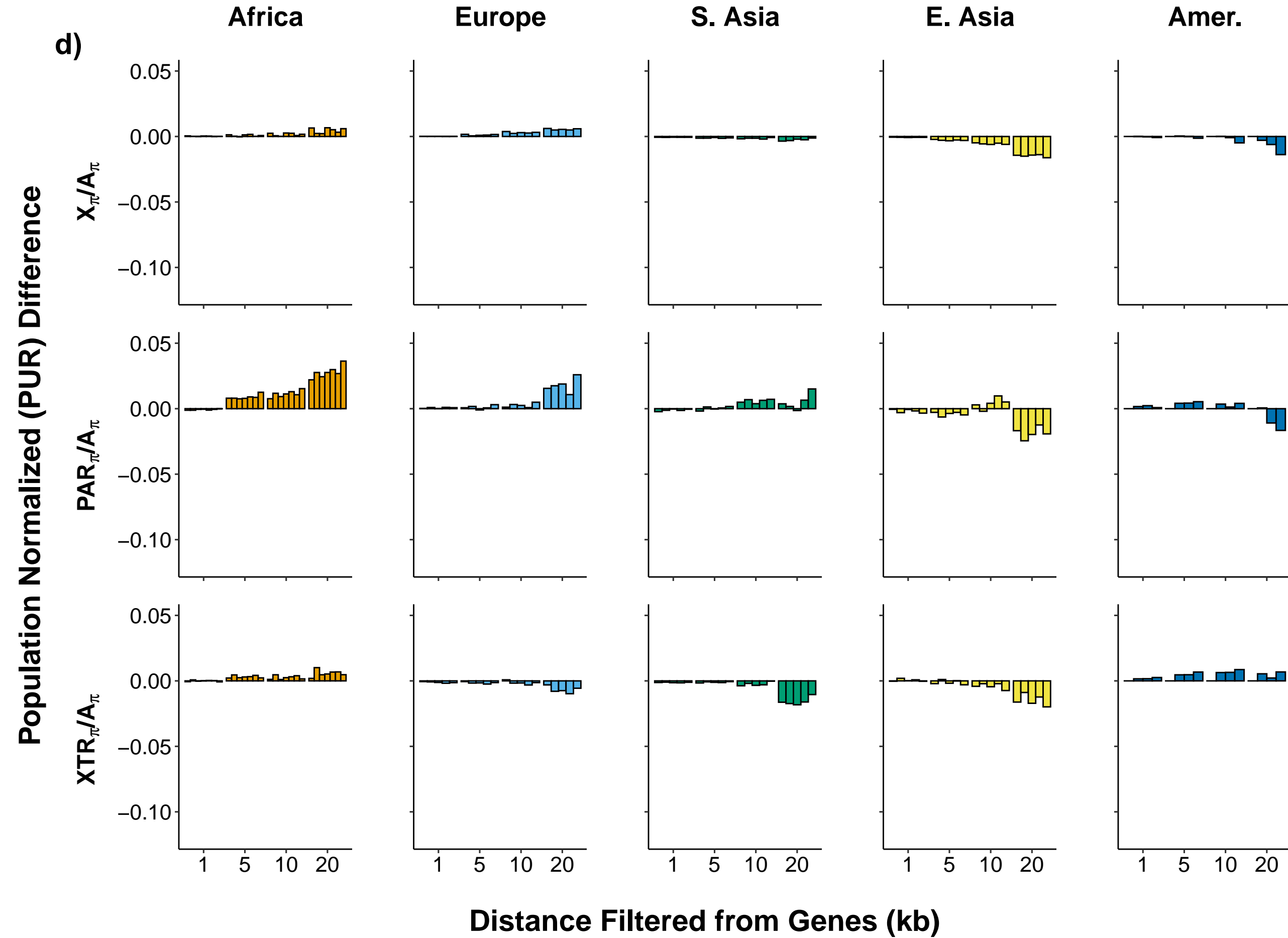

Supplement: S3 Fig — Diversity ratios between regions on the X chromosome—non-pseudoautosomal X (X), pseudoautosomal region 1 (PAR), and X-transposed region (XTR)—and autosomes for 25 1000 genomes populations. Values are reported as the difference between using a filter for only genes and a filter including 1kb, 5kb, 10kb, and 20kb of sequences flanking genes. These ratios are demography normalized by reporting each population relative to a) Toscani in Italia; b) Punjabi from Lahore, Pakistan; c) Kinh in Ho Chi Minh City, Vietnam; and d) Puerto Ricans from Puerto Rico. The order of populations is the same as reported in Fig 1B (less the corresponding population used for the correction). (PDF) [file pone.0287609.s003.pdf]

rheMac2

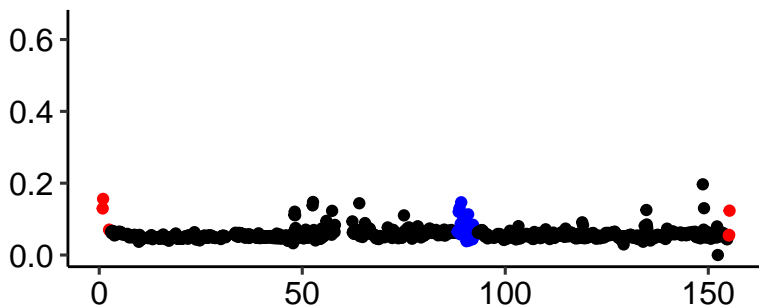

calJac3

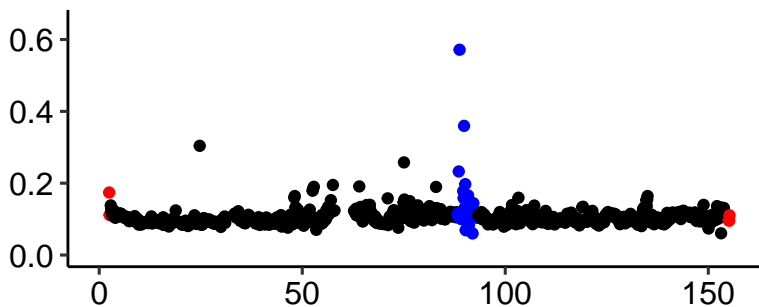

canFam3

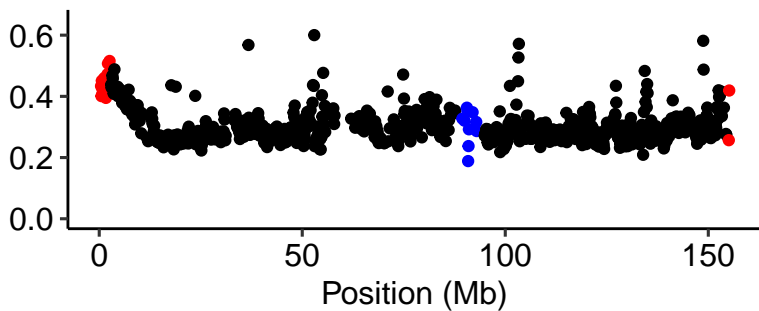

Supplement: S4 Fig — Substitution rates calculated in 100kb sliding windows and corrected using the Jukes-Cantor 1969 model [89] across the human X chromosome between the human reference (hg19) and Rhesus macaque (rheMac2; top), Callithrix jacchus (calJac3; middle), and Canis lupus familiaris (canFam3; bottom). Red indicates the pseudoautosomal regions (PAR1, PAR2) and blue indicates the X-transposed region (XTR). (PDF) [file pone.0287609.s004.pdf]
